# Dependence of Brain Intravoxel Incoherent Motion Perfusion Parameters on the Cardiac Cycle

**DOI:** 10.1371/journal.pone.0072856

**Published:** 2013-08-30

**Authors:** Christian Federau, Patric Hagmann, Philippe Maeder, Markus Müller, Reto Meuli, Matthias Stuber, Kieran O’Brien

**Affiliations:** 1 Centre Hospitalier Universitaire Vaudois (CHUV) and University of Lausanne, Lausanne, Switzerland; 2 The Abdus Salam International Center for Theoretical Physics, Trieste, Italy; 3 Center for Biomedical Imaging (CIBM), University of Lausanne, Lausanne, Lausanne, Switzerland; 4 Center for Biomedical Imaging (CIBM), University of Geneva, Geneva, Switzerland; University Medical Center (UMC) Utrecht, Netherlands

## Abstract

Measurement of microvascular perfusion with Intravoxel Incoherent Motion (IVIM) MRI is gaining interest. Yet, the physiological influences on the IVIM perfusion parameters (“pseudo-diffusion” coefficient D*, perfusion fraction f, and flow related parameter fD*) remain insufficiently characterized. In this article, we hypothesize that D* and fD*, which depend on blood speed, should vary during the cardiac cycle. We extended the IVIM model to include time dependence of D* = D*(t), and demonstrate in the healthy human brain that both parameters D* and fD* are significantly larger during systole than diastole, while the diffusion coefficient D and f do not vary significantly. The results non-invasively demonstrate the pulsatility of the brain’s microvasculature.

## Introduction

The sensitivity of the Nuclear Magnetic Resonance (NMR) signal to nuclei motion has been well documented [1], [2]. The standard Hahn spin echo sequence [1] refocuses the dephasing of spins due local field inhomogeneities with a 180° radio frequency pulse. Stejskal and Tanner [3] noted that when a pair of gradient pulses are placed either side of the 180° radio frequency pulse, any movements of a spin during these gradients leads to residual phase offsets that effect the amplitude of the measured echo. The type of movement that occurs can be investigated by varying the amplitude and direction of the two gradient pulses [4]. Magnetic Resonance Imaging (MRI) can exploit these principles to measure translational [5] and random motion, which can be measured with Intravoxel Incoherent Motion (IVIM) MRI [6], through the application of diffusion-weighted gradients followed by an Echo-Planar Imaging (EPI) signal readout [7].

In biological tissue, random motion can arise from various compartments [8], including the microvascular compartment [9]. Various models that take into account this microvascular dependence have been proposed [9]–[12], which have different underlying assumptions for the microvascular topology and the dependence between blood speed and the parameters of the MRI diffusion sequence [13]. The most common approach is to model the signal amplitude decay as function of the b-value as a bi-exponential decay [9]. This IVIM model permits the extraction of three microvascular perfusion parameters: the perfusion fraction f, which is related to blood volume; the pseudo-diffusion coefficient D*, which corresponds to the “apparent” diffusion of the microvascular compartment and is related to blood speed; and the product of f and D*, fD*, which is related to microvascular blood flow [14].

Recently, encouraging results using this model have been obtained in various pathologies in the body [15] and in the brain [16]. Nevertheless, studies validating the IVIM method to measure perfusion are rare. In the pancreas, the IVIM perfusion parameters nearly vanished under blood suppression [17]. In the kidney, a correlation between f and phase-contrast flow has been demonstrated over the cardiac cycle [18]. In the brain, a gradual increase of the IVIM perfusion parameters under a gradually increased hypercapnia challenge, known to increase cerebral perfusion, has also been demonstrated [19]. Thus, the purpose of this study was to gain further insight into the nature of the IVIM perfusion parameters in the brain, by assessing their dependance on the cardiac cycle.

## Materials and Methods

### IVIM Model

The IVIM model (**Equation 1**) was proposed by Le Bihan et al [9] to explain the bi-exponential decay of signal amplitude as function of b-value (for b<1000 mm^2^·s^−1^, defined in **Equation 2**), observed when a Stejskal-Tanner [3] diffusion sequence is applied to a biological tissue. The model proposes that the signal arises from two physically distinct compartments, a “vascular” and a “non-vascular” compartment, each with different exponential decay rates. The vascular compartment is assumed to be made of a sufficiently dense, isotropic, microvascular network, so that the movement of blood through this network presents statistical properties that can be described macroscopically with a “pseudo”-diffusion coefficient D*. The fraction of signal arising from the vascular compartment is called perfusion fraction f, and the (apparent) diffusion coefficient observed in the non-vascular compartment is called the diffusion coefficient D. For perfusion to make physiological sense, one expects D*>D.



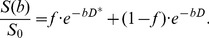
(1)S(b) and S_0_ represent the signal obtained at a given b-value and with no diffusion gradient applied, respectively. The b-value is dependent on the gyromagnetic ratio (γ), duration (δ), strength (G) and time interval (Δ) between the pair of diffusion-weighted gradient pulses:



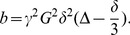
(2)Importantly for this study, **Equation 1** is still valid in the case of time dependent (pseudo)-diffusion, by assuming an effective (pseudo)-diffusion, obtained by averaging over the diffusion time Δ, as shown in the **Appendix S1**.

### Human Subjects

This prospective study was approved by the local ethics committee at the University Hospital in Lausanne (Commission cantonale (VD) d'éthique de la recherche sur l'être humain). Informed written consent was obtained from all participants. Imaging was performed on 20 healthy subjects without known history of disease (10 men and 10 women, mean age 24), from November 2011 to April 2012. No subject had to be excluded from this study.

### Imaging Parameters

**Figure 1:**
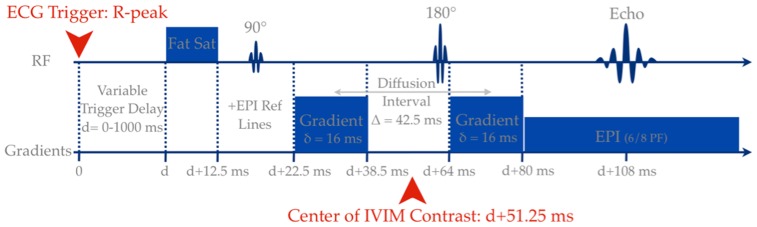
Schematic representation of the temporal position of the various gradients and pulses of the Stejskal-Tanner EPI sequence used, with respect to the cardiac trigger.

MRI-compatible electrocardiogram electrodes were placed on the chest of the subjects. Data were acquired on a 3 Tesla MR scanner (Trio, Verio, or Skyra; Siemens, Erlangen, Germany) using a 32-multichannel receiver head coil with a prospectively triggered Stejskal-Tanner diffusion weighted EPI sequence [3], [7] (**Figure 1**). For each subject, a single axial brain slices of 4 mm thickness was acquired with an in plane resolution of 1.2×1.2 mm^2^. Variation in the subject’s heart rate leads to a variable TR; however a minimum TR of 5000 ms was applied to minimize the unwanted signal variations due to T1 relaxation. The TE was minimized on each scanner (88–92 ms) with parallel imaging acceleration factor of 2, and 75% phase partial Fourier. Fat was suppressed with a frequency selective saturation pulse. Images were acquired at multiple b values (0, 10, 20, 40, 80, 110, 140, 170, 200, 300, 400, 500, 600, 700, 800, 900 s/mm^2^) in 3 orthogonal directions, by varying the amplitude of the diffusion weighted gradient, while the duration and time interval between the pulses was kept constant. The diffusion weighted images from each of the 3 orthogonal directions were subsequently combined to form the trace, removing any directional dependence.

To adequately cover both systole and diastole, repeated measurements were acquired at delays of 0, 100, 200, 300, 400, 600 and 800 ms in all subjects. In a sub-set of 11 subjects a non-gated measurement was also acquired for comparison with a standard IVIM protocol. Finally in a further sub-set of 5 subjects, additional measurements were acquired during systole for comparison against the blood flow of a feeding artery. These phase contrast flow measurements were acquired in the anterior cerebral artery with a retrospectively gated phase-contrast sequence (TR 14 ms, TE 3.14 ms, Venc 100 cm/s, Voxel size 1.0×1.4×5.0 mm, 40 frames) and analyzed using Argus (Syngo Via, Siemens, Erlangen, Germany).

### Processing and Analysis

**Figure 2:**
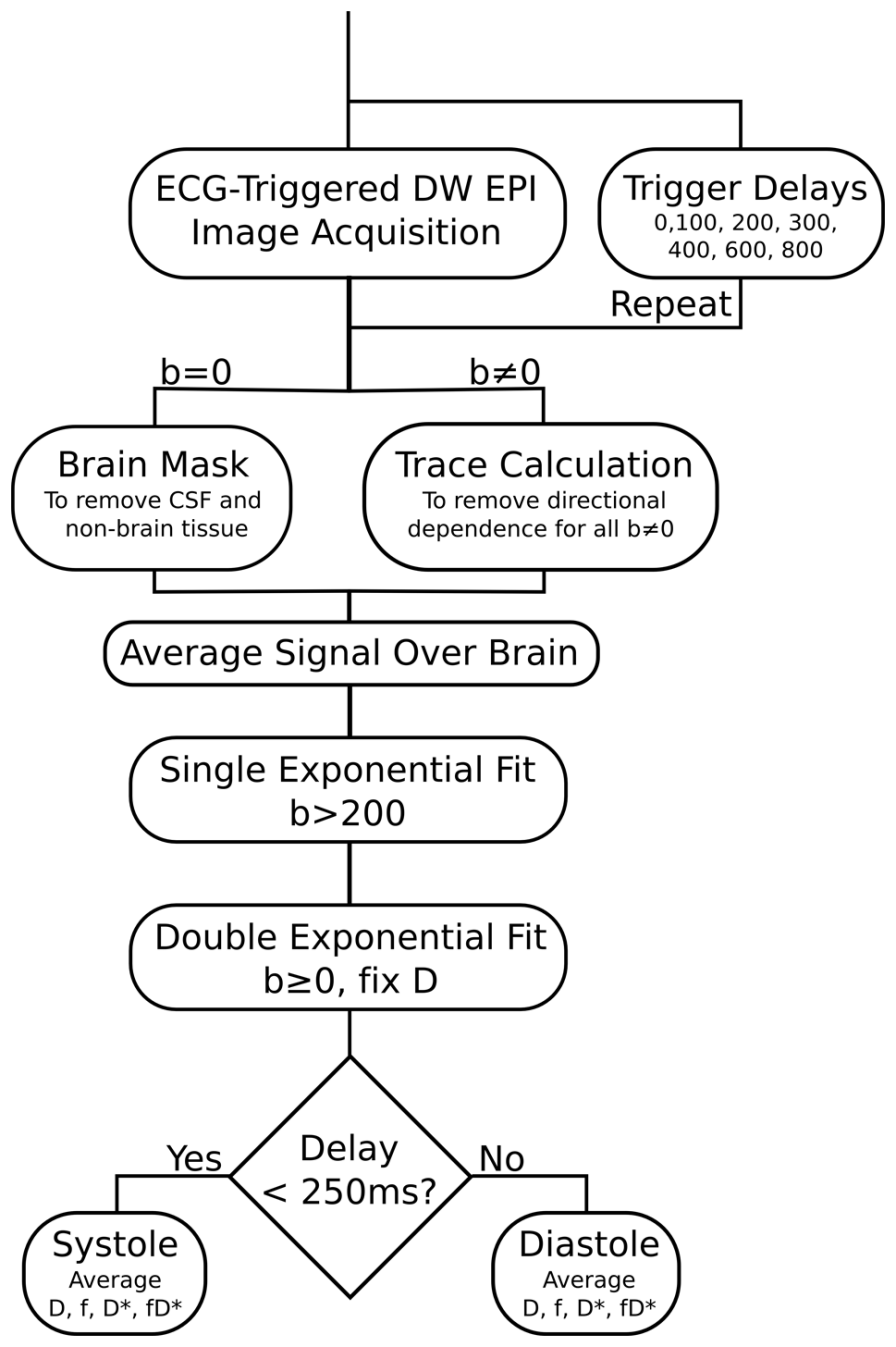
Flow chart showing the steps of the quantitative analysis algorithm.

All post processing of IVIM data was performed offline using customized Matlab scripts (Mathworks, Natick, MA). A ROI was first drawn over a full axial brain slice, from which the signal from the cerebral spinal fluid (CSF) was excluded by a threshold on the b = 0 image, which was corrected manually if required (**Figure 2**). The signal obtained was then averaged over all voxels. If we assume that D* is significantly greater than D, then for b-values greater than 200 s/mm^2^ the influence of pseudo-diffusion on the signal decay can be neglected [20]. **Equation 1** thus simplifies to a single exponential decay allowing the parameter D to be estimated. f and D* was then estimated by fitting **Equation 1** over all b-values, while keeping D constant.

Parametric maps were produced following the fitting method described above, but on a voxel-by-voxel basis. Values under 0 for f, D, and D* are not physiological and were set to 0. Values with f >0.3 and D*>0.12 mm^2^/s were also set to zero since they are also not physiological and are likely to result either from noise or turbulent CSF flow.

Systole was defined in the brain as the first 250 ms of the cardiac cycle. Mean systolic f, D, D* and fD* were obtained by averaging measurements at delays of 0, 100 and 200 ms whilst mean diastolic f, D, D* and fD* were obtained by averaging measurements at delays of 300, 400, 600 and 800 ms.

To compare against the phase contrast flow measurement, the temporal center of the IVIM sequence was defined to be in the center of both Stejskal-Tanner gradients, which is the point where we expect the maximum IVIM effect if a symmetric time dependent (pseudo)-diffusion coefficient is assumed (**Appendix S1**). Neglecting the delay between the R-peak and the trigger initializing the scan, this was determined to be ∼50 ms after the ECG Trigger.

### Statistical Analysis

Bland Altman analysis and Student’s paired T-test was performed with Matlab to compare the IVIM parameters in systole and diastole for all 20 subjects and between systole, diastole and the non-gated acquisition in the sub-group of 11 subjects. The Bonferroni correction was used to correct for multiple comparisons (f, D, D*, and fD*), thus the statistical significance to reject the null hypothesis was set to p<0.0125.

## Results

### fD* and D* were Significantly Increased during Systole

**Figure 3:**
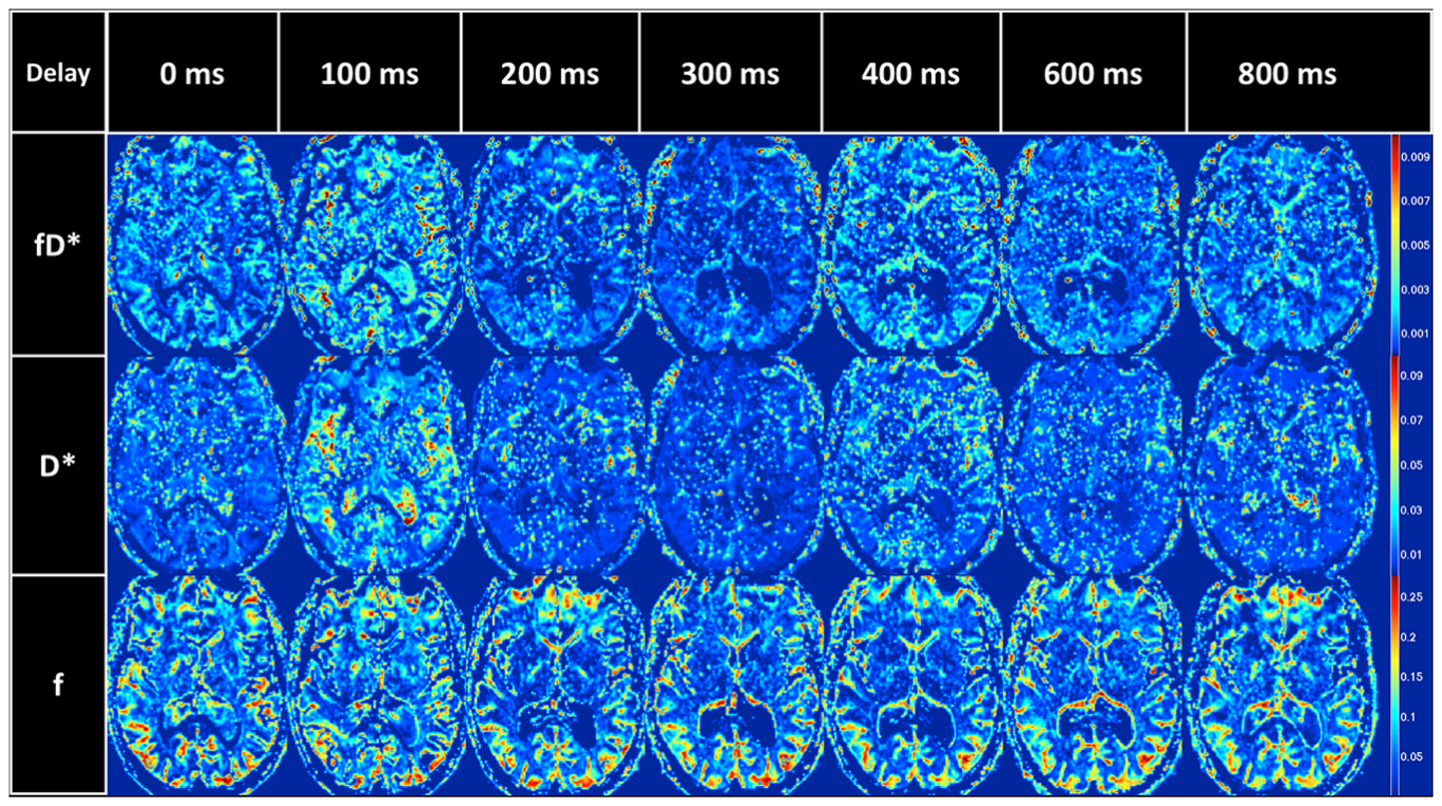
IVIM parametric maps of the blood flow related fD*, the pseudo-diffusion coefficient D*, and the perfusion fraction f, of an axial brain slice in a single subject, acquired at various delays in the cardiac cycle.

**Figure 4:**
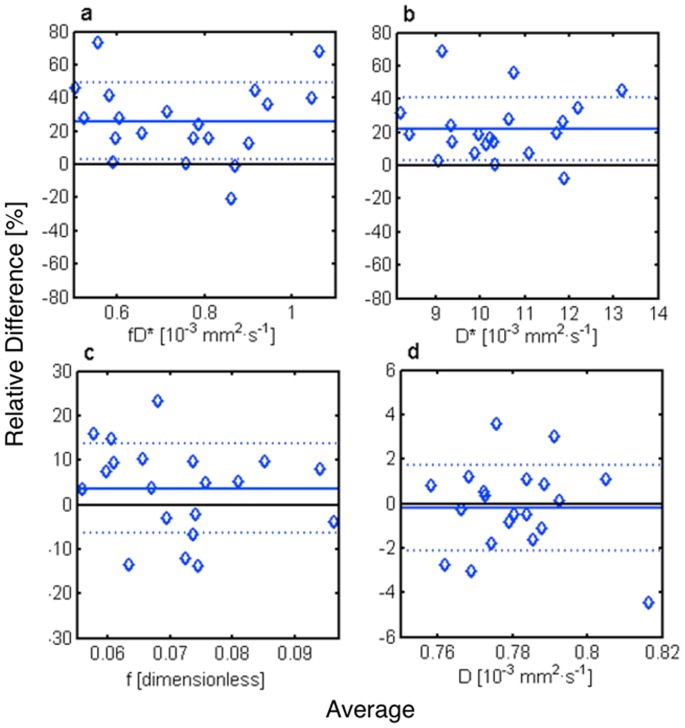
Bland Altman plots showing the relative difference between systole and diastole vs. their average for the blood flow related fD* (a), the pseudo-diffusion coefficient D* (b), the perfusion fraction f (c), and the diffusion coefficient D (d). The mean relative difference (straight line) ± standard deviation (dotted line) were fD* = 26.1±23.0%, D* = 22.0±18.6%, f = 3.6±10.1% and D = −0.2±1.9%.

The measured IVIM perfusion parameters fD* and D* were shown to vary as a function of the time delay after the R-peak of the ECG (**Table 1** **,** **Figure 3**). The Bland Altman analysis (**Table 2** **,** **Figure 4**) showed a statistically significant (p<0.0125) increase in pseudo-diffusion D* of 22.0±18.6% and in flow related parameter fD* of 26.1±23.0% between systole and diastole. In comparison, there was no statistical evidence of a difference in perfusion fraction f (3.6±10.1%) or diffusion D (−0.2±1.9%).

**Table 1:** Averaged blood flow related fD*, pseudo-diffusion coefficient D*, perfusion fraction f, and diffusion coefficient D, as measured over an entire axial brain slice in 20 subjects, after various delays after the cardiac systole (R-peak).

Delay	fD* [10^−3^ mm^2^·s^−1^]	D* [10^−3^ mm^2^·s^−1^]	f [%]	D [10^−3^ mm^2^·s^−1^]
**0**	0.74±0.41	11.08±6.10	6.74±3.21	0.77±0.02
**100**	1.02±0.49	12.86±4.02	7.84±1.20	0.78±0.02
**200**	0.92±0.15	12.28±4.51	7.36±1.69	0.77±0.02
**300**	0.64±0.23	9.02±1.70	7.14±0.67	0.77±0.02
**400**	0.67±0.12	9.41±2.01	7.04±1.42	0.78±0.02
**600**	0.76±0.12	10.29±1.11	7.49±1.40	0.78±0.02
**800**	0.70±0.25	9.81±1.99	7.13±1.58	0.78±0.02
**n.g.**	0.63±0.15	8.80±1.23	7.13±1.12	0.77±0.01

Data are averages ± standard deviation; n.g. = non-gated.

**Table 2:** Bland Altman comparison, average μ ± standard deviation σ, bias and p-values, between systolic and diastolic IVIM measurements (n = 20).

	Systole (μ ± σ)	Diastole (μ ± σ)	Bias (μ ± σ)	Paired t-test
fD* [10^−3^ mm^2^·s^−1^]	0.85±0.22	0.66±0.16	0.20±0.19	0.0003
D* [10^−3^ mm^2^·s^−1^]	11.5±1.8	9.2±1.4	2.3±2.0	0.0001
**f [%]**	7.3±1.1	7.0±1.2	0.3±0.7	0.15
**D [10^−3^ mm^2^·s^−1^]**	0.78±0.01	0.78±0.02	−0.002±0.015	0.65

### Non-gated IVIM Parameters were Similar to Diastolic Parameters

No statistical evidence of difference was found between IVIM parameters acquired without gating and IVIM parameters acquired during diastole (**Table 3**). Yet systolic pseudo-diffusion D* was still found to have a statistically significant increase compared to the non-gated acquisition (25.5±17.2%; p<0.0125). The flow related parameter fD* showed some evidence of an increase in systole (24.6±25.8%; p = 0.024) whilst the other parameters showed no evidence of a change.

**Table 3:** Bland Altman comparison, average μ ± standard deviation σ, bias and p-values, between non-gated (n.g.) and respectively systolic and diastolic IVIM measurements (n = 11).

	Non gated(μ ± σ)	Systole (μ ± σ)	Diastole (μ ± σ)	Bias n.g. - systole (μ ± σ)	Paired t-test n.g. - systole	Bias n.g. - diastole(μ ± σ)	Paired t-test n.g. - diastole
fD* [10^−3^ mm^2^·s^−1^]	0.63±0.14	0.81±0.22	0.59±0.15	0.18±0.22	0.024	−0.04±0.14	0.37
D* [10^−3^ mm^2^·s^−1^]	8.8±1.2	11.4±1.2	8.9±1.5	2.6±1.7	0.0007	0.082±1.26	0.84
**f [%]**	7.1±1.1	7.0±1.1	6.5±1.1	−0.1±0.9	0.65	−0.6±0.8	0.06
**D [10^−3^ mm^2^·s^−1^]**	0.78±0.01	0.78±0.01	0.78±0.01	0.002±0.016	0.65	−0.0001±0.01	0.97

### Comparison of IVIM Blood-flow Related fD* and Peak Flow in the Anterior Cerebral Artery (ACA)

**Figure 5:**
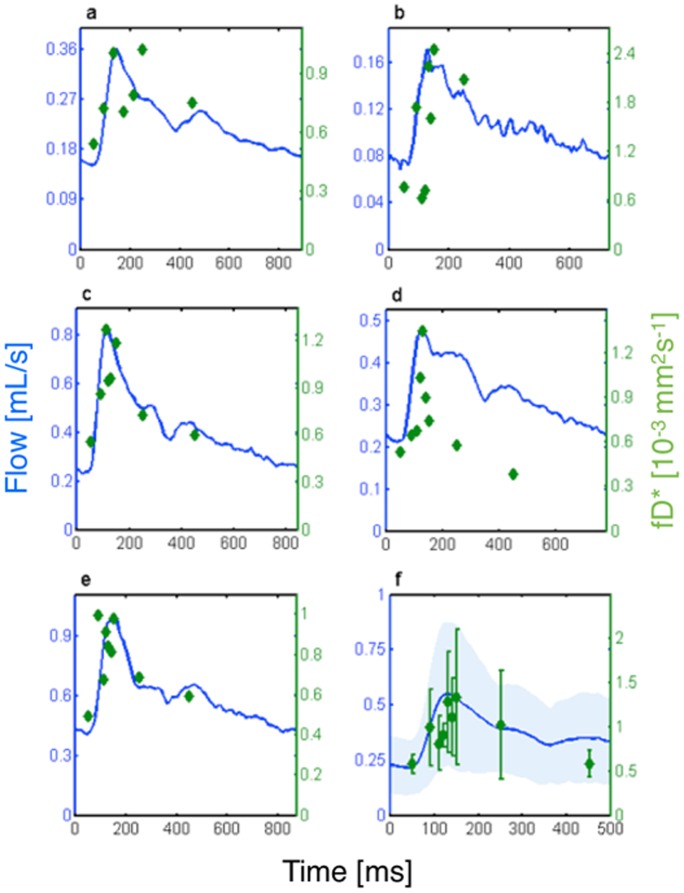
Phase contrast flow measurement (blue) and blood flow related parameter, fD*, as function of the cardiac cycle for five subjects (a–e). (**f**) Group average (errorbars give ± standard deviation) of fD* overlaid on a group average of flow measurements in the ACA (shading gives ± standard deviation).

**Figure 5** qualitatively compares the flow related parameter fD*, with the phase contrast flow measurement in the ACA as a function of the cardiac cycle for each of the five subjects (**Figure 5** **a–e**). In each subject, a systolic upstroke of the fD* parameter is observed, which agrees well with the systolic upstroke of the flow measurement. Though more noisy, a downstroke of the fD* parameter is also observed and agrees with the return of the flow measurement to baseline during diastole. In the group average (**Figure 5** **f**), fD* showed a similar global time dependence to the flow in the ACA, with a slightly delayed time to peak (∼130 ms vs ∼120 ms).

## Discussion

We observed a statistically significant increase of D* and fD* during systole. Under the IVIM model this can be interpreted as an increased blood speed or perfusion in the microvasculature and provides experimental evidence of pulsatile flow in the human brain microvasculature. No significant increase in perfusion fraction f could be found, indicating no significant rise in microvascular blood volume during the passage of the pressure wave. The stability of D during the cardiac cycle demonstrates the expected independence of the diffusion coefficient in the non-vascular compartment. IVIM perfusion parameters obtained without cardiac gating were similar to those obtained during the later part of the cycle, probably because diastole is much longer than systole, and therefore, a random measure is more likely to occur in diastole.

In humans, pulsatile flow in the microvasculature has to our knowledge only been observed in precapillary arterioles of the human conjunctiva [21] and in capillaries of the skin [22], but has been demonstrated in many animal experiments [23]–[27]. In the brain, measurements were technically limited to the surface pial vessels of rats [28] and mice [29]. A velocity pulse was observed, while no changes in the width of vessels could be detected, correlating well with our results in the human. Observation in deeper layers of tissue were recently possible with the help of two-photon microscopy in the cortex of rats [30] and mice [31] brains and showed heartbeat-induced speed modulation of red blood cells in nearly all vessels of the cortex including capillaries and venules.

The importance of the pulse has remained controversial throughout the history [32], [33]. Pulsatile perfusion has been shown to be beneficial during cardiopulmonary bypass or isolated organ perfusion [33], but numerous studies show contradicting effects on mortality, myocardial infarction, stroke, or renal failure [34]. Pulsatile flow might increase perfusion. Indeed, the energy needed to create pulsatile flow has been shown to be higher than to create non-pulsatile flow of identical mean pressure [35]. This extra energy is thought to dissipate and therefore maintain peripheral perfusion by keeping capillary beds open. It has for example been shown that pulsatility improves hemodynamics during fetal bypass, by preventing the progressive rise in peripheral and placental vascular resistances observed during steady flow fetal bypass and leading to progressive irreversible hypoxemia [36]. Therefore, pulsatility might be of importance for brain function and neural tissue health.

There are several limitations to our study. First, the duration of the IVIM sequence puts a limit to the temporal resolution of the experiment, especially the diffusion gradients, whose duration are not infinitesimal and blur the time scale. Second, the signal from the CSF, which is known to be turbulent, although carefully removed by thresholding, could still have affected the results through partial volume effects. Third, brain pulsation (i.e. of white and gray matter) itself, instead of pulsation of the vascular compartment, could have been measured, but this seems not very plausible, because a variation of the parameter f during the cardiac cycle would have been expected. Further, a significant contribution from turbulence in the vessels is unlikely, due to the low Reynolds [37]–[39] and Wormsely numbers [39]–[40] of arterioles and small arteries, ensuring mainly laminar flow. Finally, the definition of the temporal center of the IVIM pulse sequence is not trivial. In this report, it has been defined as the midpoint of the diffusion interval. As derived in the **Appendix S1**, this accurately maximizes the signal drop if the time dependent pseudo-diffusion-coefficient D*(t) is symmetric with respect to the blood flow peak time. A non-symmetric D*(t) would displace the IVIM temporal center.

## Conclusion

We extended the IVIM model to include a time dependent pseudo-diffusion coefficient, and present new insight into the nature of the IVIM perfusion parameters in the brain, by demonstrating their dependances on the cardiac cycle. It offers a new way to study the microvascular pulsatility non-invasively, on which little is known. Microvascular pulsatility could be an important factor in diseases such as cerebrovascular diseases or systemic hypertension.

## Supporting Information

Appendix S1 **Time Dependent Diffusion in Pulsed Gradients.**
(PDF)
