# Supplementary material for: Dependence of Brain Intravoxel Incoherent Motion Perfusion Parameters on the Cardiac Cycle
Source: PLoS One. 2013 Aug 30;8(8):e72856. doi: 10.1371/journal.pone.0072856 (PMC3758329; doi:10.1371/journal.pone.0072856)
Supplement: Appendix S1 — Time Dependent Diffusion in Pulsed Gradients. (PDF) [file pone.0072856.s001.pdf]

Time Dependent Diffusion in Pulsed Gradients

We consider diffusing particles with time-dependent pseudo-diffusion constant $D^*(t)$, as arises, e.g., in a fluid pushed through a randomly bifurcating network of capillaries with time-dependent speed. The particles are subject to magnetic field diffusion gradients $g(t)$. The fraction of spin echo signal with and without diffusion gradients is

$$\frac{S(g(t),t)}{S_0} = \left|\overline{e^{i\phi_j(t)}}\right|^2, \tag{1}$$

where

$$\phi_j(t) = \gamma \int_0^t g(t')r_j(t')dt' = \gamma \int_0^t g(t') \int_0^{t'} v_j(t'')dt''dt' \tag{2}$$

is the phase accumulated by particle j, and $\overline{(...)}$ is the ensemble average over the particles j, whose co-ordinate r_j undergoes pseudo-diffusion.

ϕ is a Gaussian variable, since the particle velocities $v_j(t)$ are Gaussian, satisfying

$$\overline{v_j(t)v_j(t')} = D^*(t)\delta(t-t'), \tag{3}$$

and

$$D^*(t) \approx v_{\mathrm{blood}}(t) l_{\mathrm{cap}}, \tag{4}$$

where l_{cap} is a typical length of a capillary segment between two bifurcations and $v_{\mathrm{blood}}(t)$ is the instantaneous speed of the flow within a capillary.

It is then straightforward to evaluate the Gaussian

$$\frac{S(g(t),t)}{S_0} = \exp\left[-\overline{\phi_j(t)^2}\right] = \exp\left[-\gamma^2 \int_0^t dt' \int_0^t dt'' g(t')g(t'') \int_0^{\min(t',t'')} dt''' D^*(t''')\right]. \tag{5}$$

In the special case of the Stejskal-Tanner sequence, $g(t)$ consists of two short pulses of duration δ, the first one around t_1 with amplitude $G > 0$, and the second one around $t_2 \equiv t_1 + \Delta$ with amplitude $-G$. In this case one obtains

$$\frac{S(t_1)}{S_0} \approx \exp\left[-\gamma^2\delta^2G^2 \int_{t_1}^{t_1+\Delta} D^*(t)dt\right]. \tag{6}$$

This is minimized by maximizing the value of $\int_{t_1}^{t_1+\Delta} D^*(t)dt$. If $D^*(t)$ is symmetric with respect to the blood flow peak time t_0, the maximum dephasing will be achieved when t_1 and $t_1 + \Delta$ are chosen symmetrically with respect to t_0 as well. If D^* is constant and δ is very short, the exponent reduces to Eq. (2) in the main text. For each time $\tilde{t}$, an effective $D^*_{\mathrm{eff}}(\tilde{t})$ can be obtained by averaging $D^*(t)$ over Δ:

$$D^*_{\mathrm{eff}}(\tilde{t}) = \frac{1}{\Delta} \int_{\tilde{t}-\frac{\Delta}{2}}^{\tilde{t}+\frac{\Delta}{2}} D^*(t)dt. \tag{7}$$
